# A Partial Gene Deletion of *SLC45A2* Causes Oculocutaneous Albinism in Doberman Pinscher Dogs

**DOI:** 10.1371/journal.pone.0092127

**Published:** 2014-03-19

**Authors:** Paige A. Winkler, Kara R. Gornik, David T. Ramsey, Richard R. Dubielzig, Patrick J. Venta, Simon M. Petersen-Jones, Joshua T. Bartoe

**Affiliations:** 1 Department of Small Animal Clinical Sciences, College of Veterinary Medicine, Michigan State University, East Lansing, Michigan, United States of America; 2 Genetics Program, Michigan State University, East Lansing, Michigan, United States of America; 3 Cummings School of Veterinary Medicine, Tufts University, North Grafton, Massachusetts, United States of America; 4 The Animal Ophthalmology Center, Williamston, Michigan, United States of America; 5 Comparative Ocular Pathology Laboratory of Wisconsin, School of Veterinary Medicine, University of Wisconsin-Madison, Madison, Wisconsin, United States of America; University of Iowa, United States of America

## Abstract

The first white Doberman pinscher (WDP) dog was registered by the American Kennel Club in 1976. The novelty of the white coat color resulted in extensive line breeding of this dog and her offspring. The WDP phenotype closely resembles human oculocutaneous albinism (OCA) and clinicians noticed a seemingly high prevalence of pigmented masses on these dogs. This study had three specific aims: (1) produce a detailed description of the ocular phenotype of WDPs, (2) objectively determine if an increased prevalence of ocular and cutaneous melanocytic tumors was present in WDPs, and (3) determine if a genetic mutation in any of the genes known to cause human OCA is causal for the WDP phenotype. WDPs have a consistent ocular phenotype of photophobia, hypopigmented adnexal structures, blue irides with a tan periphery and hypopigmented retinal pigment epithelium and choroid. WDPs have a higher prevalence of cutaneous melanocytic neoplasms compared with control standard color Doberman pinschers (SDPs); cutaneous tumors were noted in 12/20 WDP (<5 years of age: 4/12; >5 years of age: 8/8) and 1/20 SDPs (*p*<0.00001). Using exclusion analysis, four OCA causative genes were investigated for their association with WDP phenotype; *TYR, OCA2, TYRP1* and *SLC45A2. SLC45A2* was found to be linked to the phenotype and gene sequencing revealed a 4,081 base pair deletion resulting in loss of the terminus of exon seven of *SLC45A2* (chr4∶77,062,968–77,067,051). This mutation is highly likely to be the cause of the WDP phenotype and is supported by a lack of detectable *SLC45A2* transcript levels by reverse transcriptase PCR. The WDP provides a valuable model for studying OCA4 visual disturbances and melanocytic neoplasms in a large animal model.

## Introduction

**Figure 1:**
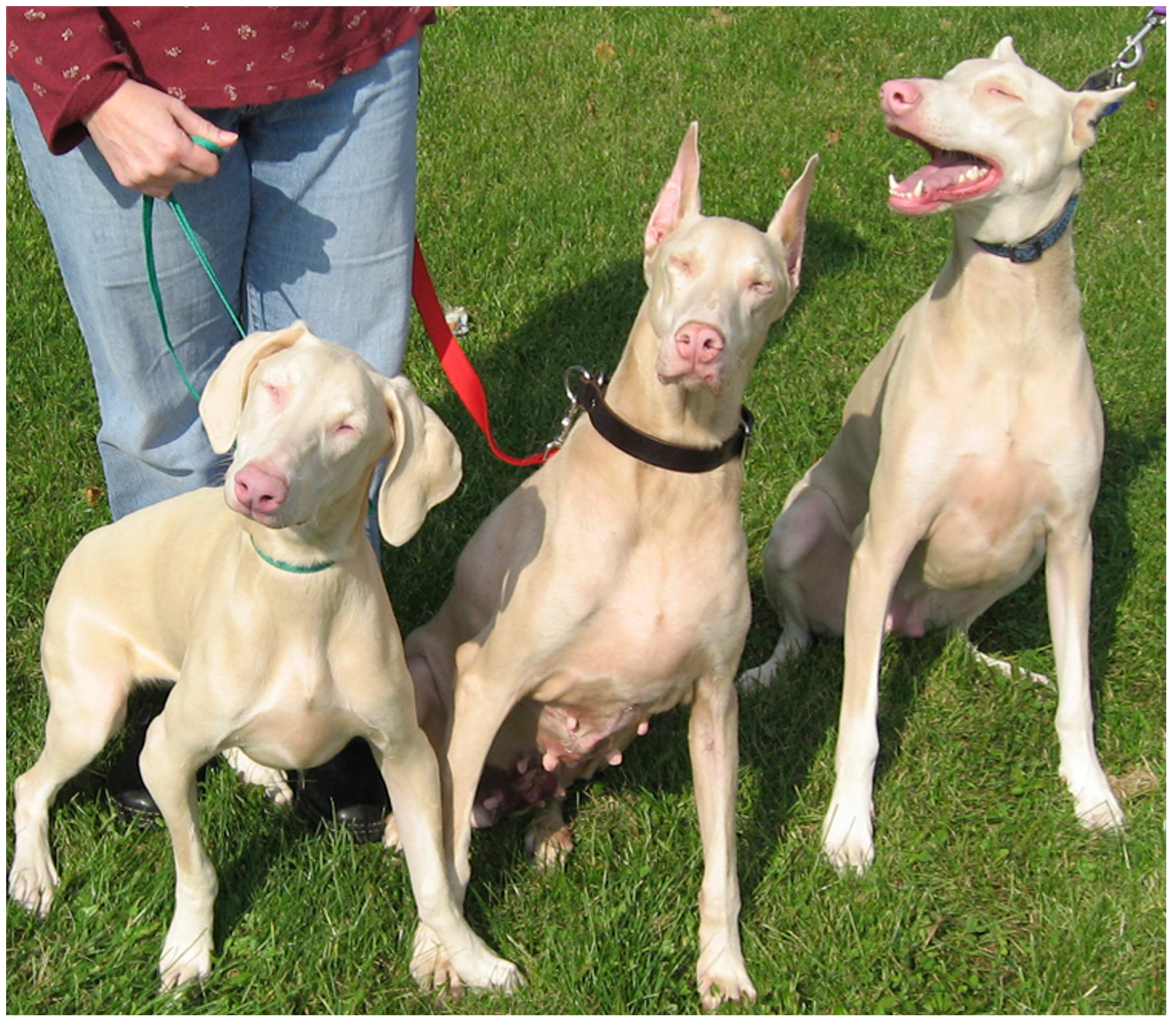
Typical appearance of white Doberman pinschers. A group of three white Doberman pinschers demonstrate the typical cream coat-coloration. Note that all three dogs are squinting in the bright sunlight; photophobia was observed in all WDPs examined for this study.

The Doberman pinscher dog breed was originally developed in the 1880s by Karl Dobermann of Thüringen, Germany, for the purpose of protection in his job as a tax collector and night watchman. Today, the Doberman pinscher is used for many other purposes, including companionship (DPCA.org). Foundation breeding dogs were imported to the United States in the early 20^th^ century. As part of the official breed standard, major international breed clubs, including the Doberman Pinscher Club of America (a member of the American Kennel Club), recognize only coat colors of black, blue, red, and fawn. In 1976, a female Doberman pinscher with novel “white” coat-coloration was born and registered with the American Kennel Club. The uniqueness of the light coat-color prompted breeders to utilize line breeding to maintain the phenotype, resulting in an extensive pedigree in which all living white Doberman pinschers (WDPs) are traceable to this initial white female. Although the coat-color was originally termed “white,” in actuality it is a light cream color (see Figure 1). Subsequent suspicion arose that the phenotype was actually a form of albinism, setting off a controversy among Doberman pinscher breeders surrounding breeding recommendations for WDPs.

Solid, or nearly solid, white coat-colors are found among numerous dog breeds (e.g., Samoyeds, American Eskimo, Maltese, and Bichon Frises) for which the causative genes have no apparent effect on phenotype other than the white coat. The eyes, nose, and lips tend to be darkly pigmented. The causative gene(s) producing the white coat-color in these breeds is currently unknown, although the *MC1R* and *agouti* loci may contribute to the phenotype [1]. Additional genes have been identified that cause white coat-color and variations of white pattern phenotypes in dogs, which are occasionally associated with other undesired traits such as deafness (e.g., Dalmatians, Australian shepherds and Boxers) [2]. These include genes such as *SILV*, *MITF* and *PSMB7* [3]–[5]. These genes are known to be involved in melanocyte migration during development and/or survival of melanocytes. Interaction between these genes and additional loci resulting in deafness remains unknown [1], [2]. In addition to producing pigment in skin and hair, melanocytes are found in the inner ear, where they function to control ion transport necessary for the function of the inner ear [6]. It has been shown in mouse and guinea pig models that when melanocytes are not present in the inner ear, animals are deaf [7]–[9].

Human oculocutaneous albinism (OCA) is a group of autosomal recessively inherited conditions affecting pigment production in the skin, hair, and eyes but do not affect hearing. Genetic mutations resulting in disruption of pigment synthesis within melanosomes are not typically associated with deafness. However, disorders or melanocyte migration, differentiation or survival can produce deafness in humans; as seen with mutations in *MITF* which result in Waardenburg syndrome, type 2a [10]. Additional Mendelian phenotypes found in humans are associated with white hair, these most notably include Hermansky-Pudlak and Chediak-Higashi syndromes; with the former, affected patients develop hemorrhagic diathesis and the latter, immune system dysfunction [11]. However, white Doberman pinschers are not deaf, and the phenotype in white Doberman pinschers does not appear to include a bleeding diasthesis, immune system defects, or any other traits associated with other rarer heritable conditions that include hypopigmentation as part of the phenotype.

**Figure 2:**
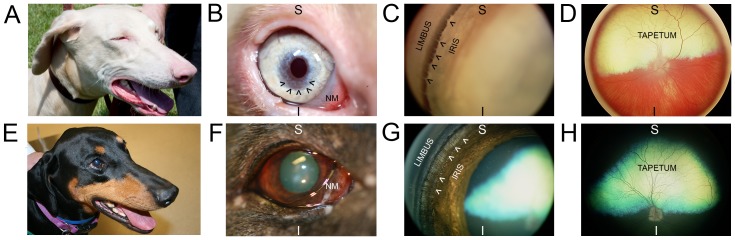
Ocular phenotype of white Doberman pinschers. Images taken from WDP (top row) and black standard-color Doberman pinscher (bottom row). An image of WDP head (A) demonstrates lightly-pigmented nose, lips, and eyelid margins compared with the same darkly pigmented structures in SDP (E). A close-up image of WDP eye (B) shows: non-pigmented leading edge of the nictitating membrane (NM), tan-colored iris base transitioning to blue at pupillary margin, and oval-shaped dyscoric pupil aperture. The black arrowheads (in B) demarcate a region of significant iridal stromal thinning that was noted on examination to transilluminate (not shown in image) with retroillumination by light reflected from the tapetum lucidum. SDP eye (F) shows: darkly pigmented margin of the nictitating membrane (NM) and brown iris with a round pupil aperture. WDP gonioscopy image (C), which allows visualization of structures lying within the iridocorneal angle (in images C & G, this region lies between the words “LIMBUS” and “IRIS”) shows fibers of the pectinate ligament (demarcated by black arrowheads) are of a similar tan-color to the iris base, whereas fibers of the pectinate ligament (demarcated by white arrowheads) are dark brown in SDP (G). WDP fundus image (D) shows yellow-colored tapetum lucidum (labeled “TAPETUM”) and significant hypopigmentation of the retinal pigment epithelium and choroid allowing visualization of the choroidal vasculature. SDP fundus image (H) shows green-colored tapetum lucidum (labeled “TAPETUM”) and heavy pigmentation of the non-tapetal fundus. For orientation purposes, images taken at higher magnification (B–D and F–H) have the superior (S) and inferior (I) globe positions labeled.

The WDP phenotype appears most similar to human OCA. WDPs have pale irides and pink noses and lips (see Figure 1 and 2 A, B). There are anecdotal reports of an increased frequency of skin tumors and nevi, as well as photophobia and vision defects in WDP (DPCA.org). A research study conducted by the DPCA concluded an autosomal recessive mode of inheritance (http://dpca.org/BreedEd/index.php/articles/44-history/381-albinism-science). Humans affected with OCA typically have very pale skin and white or light-colored hair. Long-term sun exposure greatly increases the risk of skin damage and skin cancers, including melanoma, in humans with OCA. OCA also reduces ocular pigmentation. Humans with OCA frequently have some degree of diminished visual acuity, nystagmus, and/or photophobia (http://ghr.nlm.nih.gov/condition/oculocutaneous-albinism). To date, there are seven genes/loci associated with nonsyndromic OCA in humans and other species. OCA types 1–7 are associated with the genes *TYR, OCA2, TYRP1, SLC45A2*, a locus on 4q24, *SLC24A5* and *C10orf11*, respectively [12].

In 1996, the American Kennel Club accepted a request from the Doberman Pinscher Club of America that any dog, regardless of coat-color, which contains the founding white female in the pedigree be designated by the letters ‘WZ’ in their registration number (referred to as the “Z-listing” by breeders). This allows breeders to avoid using dogs related to any WDP in their breeding lines; unfortunately the conditional registration number does not provide an indication of the actual carrier or non-carrier status of the dog.

In clinical practice two of the authors (JTB and DTR) noted a seemingly high number of periocular and intraocular pigmented masses present in WDP; ultimately further interest in this observation resulted in development of the present study. This study had three specific aims: (1) produce a detailed description of the ocular phenotype of WDP, (2) objectively determine if an increased prevalence of ocular and cutaneous melanocytic tumors was present in WDP, and (3) determine if a genetic mutation in any of the genes known to cause human OCA results in the WDP phenotype, the findings of which are reported herein.

## Materials and Methods

### Ethics Statement

All procedures were in compliance with the ARVO statement for the Use of Animals in Ophthalmic and Vision Research and approved by the Michigan State University Institutional Animal Care and Use Committee (AUF number 05-11-106-00; Institutional NIH/PHS Animal Welfare Assurance number A3955-01). Owners of WDPs and SDPs gave consent for use of their dogs in this study.

### Clinical Examinations

Applantion tonometry, slit-lamp biomicroscopy, indirect ophthalmoscopy, examination for dermal masses, and routine phlebotomy were performed as part of a standard health screening examinations by a board-certified veterinary ophthalmologist (DTR) at two regional Doberman pinscher rescue facilities; prior consent was provided by the director of each facility. Twenty white Doberman pinschers (WDPs) and twenty Doberman pinschers of breed standard coat-colors (SDPs) were evaluated. Signalment, pertinent historical information, and results of clinical examinations were collected from medical records.

### Excisional Biopsies and Histopathology

As part of routine clinical practice, three client-owned WDPs were presented to DTR for periocular, intraocular, and cutaneous mass excision. A total of six masses (4 dermal, 1 oral, 1 iridal) were excised en bloc, fixed in 10% buffered formalin and submitted to the Comparative Ocular Pathology Laboratory of Wisconsin for histopathological interpretation by one of the authors (RRD). The formalin-fixed tissue was embedded in paraffin, sectioned, stained with hematoxylin and eosin, and analyzed via light microscopy. Additionally, historical histopathology reports for five client-owned WDP presented to the clinic were obtained; the cutaneous biopsy samples (all from the facial region) had been obtained previously by referring veterinarians. Histopathologic examination of these samples was performed at the Animal Disease Laboratory-Illinois Department of Agriculture (1 case), IDEXX Laboratories (3 cases), and Kansas State Veterinary Diagnostic Laboratory (1 case).

### DNA Extraction

Blood samples were collected by one of the authors (DTR) and sent to the Comparative Ophthalmology Laboratory at Michigan State University for genetic analysis. DNA was extracted using a previously described protocol [13]. Briefly, red blood cells were hemolyzed and discarded before adding cell lysis buffer (Qiagen, Germantown, MD, USA) to lyse the white blood cells. A subsequent protein precipitation step was performed, followed by isopropanol precipitation of the DNA.

### Exclusion Analysis Using Single Nucleotide Polymorphisms and Microsatellite Markers

Based on similarity of the WDP phenotype to human OCA, four genes; *tyrosinase* (*TYR*), *P-gene* (*OCA2*), *tyrosinase-related protein 1* (*TYRP1*), *and solute carrier family, member 2* (*SLC45A2*), were selected for exclusion analysis. Exclusion analysis works under the following assumptions; (1) the underlying mutation of a high frequency Mendelian condition is identical by decent due to a founder event (i.e. no allelic or locus heterogeneity), (2) there are no phenocopies, (3) no new mutation occurs in the marker, and (4) no recombination has occurred between the marker and the candidate gene [14].

Markers were located using the RepeatMasker track (for microsatellite markers; MS) or the SNPs track on the UCSC Genome Browser 2005 build of the canine genome (http://genome.ucsc.edu/). Two markers were identified within 500 kb of either side of the gene or within the intronic regions of the gene itself. These markers were selected to be as close as possible to the gene to minimize the chance of falsely excluding the gene due to an unidentified recombination between the marker and the potential mutation within the gene.

Primers to amplify the markers were designed using Primer3 (http://bioinfo.ut.ee/primer3-0.4.0/), primer sequences and related information are given in Table 1 and Table S1. All PCR reactions consisted of 20 mM Tris-HCl (pH 8.4), 50 mM KCl, 1.5 mM MgCl_2_, 0.5 U Taq DNA polymerase (Life Technologies, Grand Island, NY, USA) and 50 ng of DNA in 20 or 25 μl reaction volumes. For PCR reactions to be used for SNP and exonic sequencing analysis, 0.3 μM of each primer was added to the PCR mix. All PCR amplifications were performed under the following conditions: 30 sec at 94°C, 30 sec at 59°C and 1 min at 72°C for 30 cycles with a final extension step of 3 min at 72°C.

**Table 1:** Markers used in exclusion analysis.

Gene	Gene Location^1^	Amplicon ID	Amplicon Location^1^	Amplicon Size^2^	Enzyme	Location^3^	Distance from gene^4^
	chr21∶13,797,070–	MS 1	chr21∶13,896,869–	398	–	U	99,799
*TYR*	13,891,317	(TTTC)	13,897,266				
		MS 2	chr21∶13,788,050–	433	–	D	103,267
*TYR*		(TTTC)	13,788,482				
	chr3∶35,208,771–	SNP 1	chr3∶35,564,467–	310	DraI	D	355,696
*OCA2*	35,559,119	rs8736779	35,564,776				
		SNP 2	chr3∶35,393,392–	213	AluI	MD	184,833
*OCA2*		rs23591920	35,393,604				
	chr11∶36,344,712–	SNP 1	chr11∶36,138,020–	118	RsaI	U	224,545
*TYRP1*	36,362,565	rs22125514	36,138,137				
		MS 1	chr11∶36,458,618–	239	–	D	113,906
*TYRP1*		(TTTC)	36,458,856				
	chr4∶77,035,754–	MS 1	chr4∶76,870,491–	325	–	U	192,640
*SLC45A2*	77,063,131	(TTTC)	76,870,815				
		MS 2	chr4∶77,081,806–	378	–	D	46,052
*SLC45A2*		(TTTC)	77,082,183				

1 Gene location and amplicon based on the UCSC Genome Browser canine reference genome, CanFam2.0. Primer sequences available in Table S1.

2 Amplicon size as predicted by the UCSC Genome Browser canine reference genome, CanFam2.0.

3 Location of the marker in respect to the gene. D, downstream of the coding regions, U, upstream of the coding regions and MD, downstream of the middle of the gene, in an intronic region.

4 Distance from the furthest end of the gene to the beginning of the marker.

Amplification products for SNP analysis were digested with an appropriate restriction enzyme (Table 1). The markers were analyzed by standard agarose gel electrophoresis using 2% agarose gels in a tris-acetate-EDTA buffer run at 200V for 45 or 60 minutes (SNP or MS, respectively).

### Sequencing SLC45A2

*SLC45A2* exons were identified using UCSC Genome Browser 2005 (CanFam2.0) canine genomic assembly. Primers were designed in the nearby intronic regions surrounding the exons (Table 2). PCR products were submitted to Michigan State University’s Research Technology Support Facility (MSU-RTSF) for Sanger dideoxy sequencing on an ABI 3730 Genetic Analyzer. Sequences were viewed using Sequencher 4.0 (Gene Codes, Ann Arbor, MI, USA).

**Table 2:** Primers used for exonic sequencing of *SLC45A2*.

Amplicon ID^1^	F Primer	R Primer	Amplicon Size^2^
Exon 1–1	TCATGCTTTCCTGACTCCAC	AATCTAGGAGAGACAATCCGTTC	716
Exon 1–2	CAACACTGGGCAGCTTGG	TCCACCGCATAGCAGAACTC	357
Exon 2	AAAGCAAAGGGAGGTAAGTTG	GGGAGCATTCCATCAACAG	403
Exon 3	TCTTGGCCTCCTGCTGTG	GAGCTTGTATGAAAGTTGTTAGAGC	626
Exon 4	CCCAGAAAGTGTGATGCTG	CCTTAAAGGTACTCTTCCTTACATTC	397
Exon 5	TGGTCATGGGAGAATGAACTC	TTTCCTGATTTCCATAATTTATCAAG	623
Exon 6	TGACTCAGCATCAAAGGAAGAC	CAGAGCTGCAGAGGAAAGAG	459
Exon 7 SDP	CAGTTTCTTGGTGACTGTAAAGC	CAGATTGTTGAGGCTGAAGTC	745
Exon 7 WDP	CAGTTTCTTGGTGACTGTAAAGC	CGTCGGTGACTGAGCAGAG	495

1 Exon 1 was too large to sequence as one product so two products were used to cover the region (Exon1–1 and 1–2). The reverse primer for sequencing Exon 7 differs between WDPs and SDPs due to the deletion.

2 Amplicon size as predicted by the UCSC Genome Browser canine reference genome, CanFam2.0.

### Estimation of the Extent of Linkage Disequilibrium around *SLC45A2*

Additional MS markers were identified in increasing distances from *SLC45A2* (Table S2). Markers were genotyped as far away as 7 Mb on either side of the gene. Primers made for MS markers used for linkage disequilibrium mapping had a universal sequence tag attached to the 5′ end of the forward primer (5′-AGGGTTTTCCCAGTCACGAC-3′) [15]. This enabled the addition of a FAM or HEX fluorescently labeled complementary primer. The chimeric forward primers were added at 0.03 μM concentration and the reverse primer and the universal primer were added at a concentration of 0.3 μM. Allele sizes were determined on an ABI PRISM 3130 Genetic Analyzer by MSU-RTSF. The results of this high resolution genotyping were analyzed with Peak Scanner software (Life Technologies, Carlsbad, CA, USA) (Table S3).

### RNA Collection and Extraction

With prior owner consent, a small skin tissue sample was collected from a larger skin sample excised from a WDP undergoing a routine surgical procedure for an unrelated medical condition at the Michigan State University-Veterinary Teaching Hospital (MSU-VTH). Following euthanasia for an unrelated study, white and pigmented skin samples were separately collected for use as control samples from a tri-colored, mixed-breed dog maintained in a research colony at MSU-VTH (AUF number 05-11-106-00). The samples were fixed in RNA Later (Qiagen, Valencia, CA, USA) and stored at −20°C until further processing. Approximately 30 mg of tissue was finely ground in liquid nitrogen using a sterile mortar and pestle. RNA was extracted following the manufacturer’s protocol (RNeasy Fibrous Tissue Kit, Qiagen, Valencia, CA, USA).

### cDNA Synthesis and Sequencing

Complimentary DNA (cDNA) was synthesized using approximately 200 ng of RNA and the components of a 3′ RACE kit utilizing a poly(dT) primer (Invitrogen, Carlsbad, CA, USA) following the manufacturer’s instructions. The resultant cDNA was stored at −20°C. A qualitative assessment of the presence/absence of *SLC45A2, TYR*, and *Beta-actin (ACTB*, selected as house-keeping control gene) expression was analyzed using a PCR product of exonic regions and compared with control skin samples. Primers were designed using Primer3 (http://bioinfo.ut.ee/primer3-0.4.0/). PCRs reactions were in 20 μl volumes as described in the “exclusion analysis” section above. Reverse transcription PCRs for *SLC45A2, TYR* and *ACTB* were performed under the following conditions: 94°C for 3 min followed by 50 cycles of 94°C for 1 min, 63°C for 2 min, and 72°C for 3 min and a final extension step of 1 cycle at 72°C for 3 min. PCR products were sent to MSU-RTSF for Sanger sequencing on an ABI 3730 Genetic Analyzer.

### Statistical Analysis

Parametrically distributed clinical examination data were compared using Student’s T-Test and non-parametrically distributed clinical examination data were compared using Chi-square analysis, with significance set at *p*≤0.05. The association of the fixed microsatellite allele in WDPs was compared with the number of alleles of that size in SDPs and analyzed for significance with a two-tailed Fisher’s Exact test. The alleles were pooled into (1) most common allele size in the WDPs and (2) all other allele sizes. The p-values reported for the *SLC45A2* markers are calculated from the allele sizes in Table S3 (192 kb upstream [U] and 46 kb downstream [D]).

## Results

### Clinical Examinations

Mean age of the WDP study population was 4.1±2.7 yr (range 0.8–11 yr) and included 11 males and 9 females. Mean age of the SDP population was 5.5±4.2 yr (range 0.4–13 yr) and included 13 males and 7 females. SDP coat-colors included: 16 black, 2 blue and 1 red.

Applanation tonometry estimated mean intraocular pressure values were 14.0±3.4 mmHg for WDPs and 13.5±3.3 mmHg for SDPs. Observation of the dogs prior to ophthalmic examinations revealed all WDPs showed photophobia outdoors in full sunlight (Figure 1), which resolved indoors in ambient room light. SDPs did not exhibit photophobia. No WDP or SDP were noted to have nystagmus. Biomicroscopy showed all WDP had hypopigmented adnexal structures including eyelid margins, leading edge of the nictitating membrane, and cilia (Figure 2 B); whereas these structures were darkly pigmented in SDPs (Figure 2 F). The irides of all WDPs were tan in the peripheral ciliary zone transitioning to blue towards the pupillary zone, the pupils showed vertically-oriented ovoid dyscoria, and had patchy stromal thinning adjacent to the pupillary aperture (Figure 2 B); these areas of stromal thinning were observed to transilluminate with retro-illumination during the ocular examinations. Irides of all SDPs were dark brown in color with round pupillary apertures (Figure 2 F) and no defects were noted with transillumination. Occasionally iridociliary cysts (WDP: 4 of 20, mean age 6.7±2.9 yr [range 3–10 yr]; SDP: 0 of 20; *p = *0.15) and incipient cortical cataracts were observed (WDP: 4 of 20, mean age 7.5±2.5 yr [range 7–11 yr]; SDP: 1 of 20, age 11; *p = *0.15). Gonioscopy showed the pecinate ligament was similar in coloration to the iridal stroma: tan in WDPs (Figure 2 C) and dark brown in SDPs (Figure 2 G). Indirect ophthalmoscopy revealed all WDP had profound hypopigmentation of the retinal pigment epithelium and choroid, making the choroidal vasculature readily apparent in the non-tapetal fundus (Fig. 2 D). In all SDPs, the retinal pigment epithelium and choroid were both heavily pigmented in the non-tapetal fundus (Fig. 2 H).

**Figure 3:**
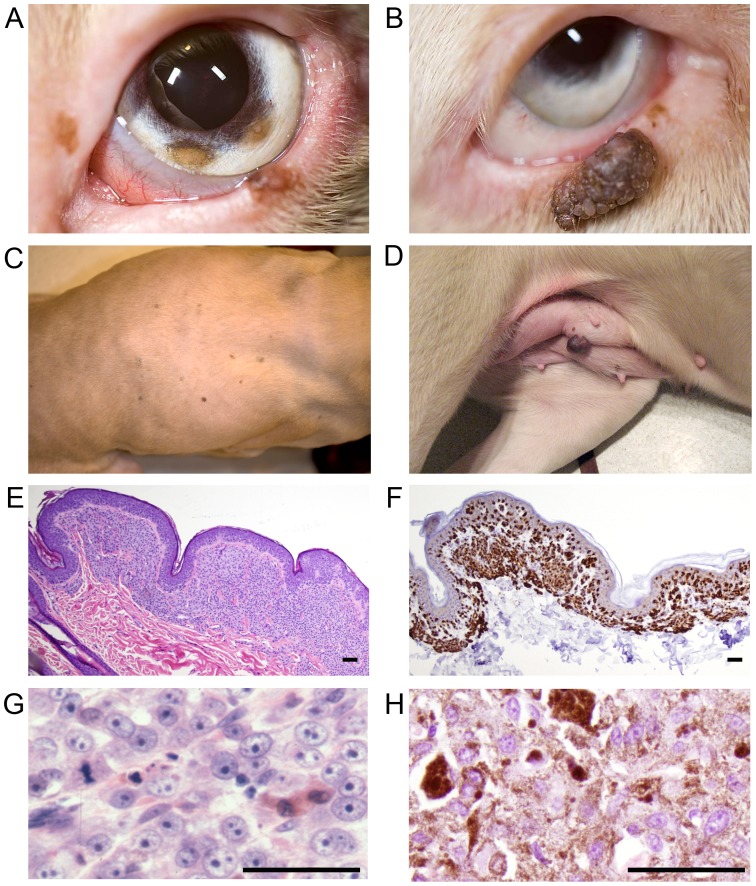
Cutaneous and ocular lesions of white Doberman pinschers. A close-up image of WDP eye (A) shows multiple pigmented nevus-like lesions on the eyelids and iris. A close-up image of another WDP eye (B) shows a large, lobulated, pigmented eyelid mass. Image of the dorsum of a WDP (C) shows numerous pigmented nevus-like lesions. Ventrum of the same WDP (D) shows a large, pedunculated, pigmented cutaneous mass. Image taken through a 4× objective microscope lens of an hematoxylin and eosin stained histopathology section of cutaneous tissue from WDP (E); note the infiltration of poorly-pigmented cells in small sheets pushed between collagen fibers and aggregated clusters within and abutting the epithelium. Immunohistochemistry with primary antibody recognizing Melan-A (melanocyte marker) from the same cutaneous biopsy sample (F); note strong labeling of the subepithelial cellular infiltrates, indicating that these poorly pigmented cells are melanocytes. Image taken through a 40× objective microscope lens of an hematoxylin and eosin stained histopathology section of a dermal mass from WDP (G) demonstrates cellular morphology of the atypical melanocytes. Although some intracellular pigment is present in (G), it is relatively sparse compared to an image taken through a 40× objective microscope lens of an hematoxylin and eosin stained histopathology section from a representative canine dermal melanoma (H) submitted to the Diagnostic Center for Population and Animal Health at Michigan State University. The amount of pigment present in (H) is considered typical for canine dermal melanoma/melanocytoma; the relative absence of pigmentation noted comparing image (G) to image (H) demonstrates why multiple examining pathologists characterized the lesions submitted from WDP as “amelanotic”. Size bars 100 um.

Careful examination of the skin revealed a significantly increased prevalence of tumors in WDPs (12 of 20, [<5 years of age: 4 of 12; >5 years of age: 8 of 8]) compared with SDPs (1 of 20, *p*<0.0001). In WDPs multiple tumors were frequently observed (10 of 20), and locations included the skin and lips (12 of 20 dogs), eyelids (10 of 20 dogs), and iris (1 of 20 dogs) (Figure 3 A–D). Tumors of WDPs ranged in size from 1 mm to approximately 20 mm, in color from light brown to dark burgundy, and in shape from flat to raised to pedunculated. The tumors on WDP were noted on both dorsal and ventral aspects of the body and the apparently random distribution did not suggest a predilection for sites with maximum ultraviolet light exposure. Only one SDP was found to have a single darkly pigmented 2 mm superior eyelid mass.

### Histopathology

Cutaneous, oral, and iridal masses examined histologically by RRD consisted of regional infiltration of melanocytic cells in small sheets pushed between collagen fibers and aggregated clusters within and abutting the epithelium (Figure 3 E). Infiltrating cells were predominantly small cells with darkly staining oval nuclei, a secondary population of larger stellate to polygonal cells, and small number of pigmented cells. All three cell populations labeled positively for Melan A by immunohistochemistry (Figure 3 F). For WDP, the diagnosis was amelanotic melanocytoma in all cases. Diagnoses on the historical biopsy reports included: multicentric malignant amelanotic melanoma, neoplasia-undetermined with consideration given to amelanotic epitheliod variant of malignant melanoma, dermal melanocytoma, cutaneous malignant melanoma-amelanotic, and malignant melanoma of haired skin. Compared to biopsy samples of cutaneous melanoma or melanocytoma from standard colored dogs, much less pigment is apparent in the histology section from WDP (Figure 3 G–H). To visualize morphology of intracellular structures in sections of cutaneous melanoma or melanocytoma from standard colored dogs, commonly bleaching of sections is necessitated; however, intracellular structures are readily apparent in high-magnification images of sections from WDP (Figure 3 G). The relative reduction in pigment visualized in unbleached sections explains why multiple examining pathologists characterized the lesions submitted from WDP as “amelanotic”.

### Exclusion Analysis

**Figure 4:**
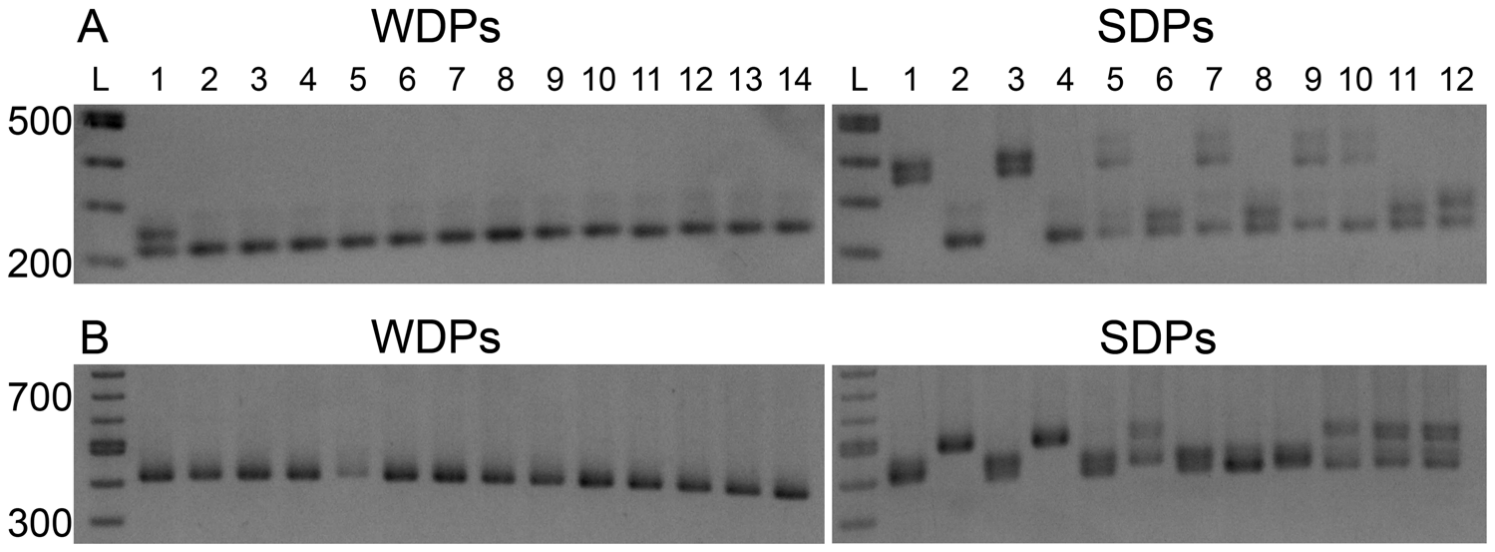
Lack of exclusion of *SLC45A2* with white Doberman pinschers as assessed by genotyping microsatellites by agarose gel electrophoresis. 2.0% agarose gel comparisons of (A) *SLC45A2* microsatellite marker 1 (192 kb upstream of *SLC45A2*), white Doberman pinschers (WDPs, left hand portions of panel A) and standard color Doberman pinschers (SDPs, right hand portion of panel A) and (B) *SLC45A2* microsatellite marker 2 (46 kb downstream of *SLC45A2*), for WDPs and SDPs. Note the high variability in the markers in the SDPs compared with the almost complete lack of variation seen for WDPs (the extra band seen in lane 1 is a heteroduplex band caused by a one-step tetranucleotide repeat mutation [see Table S2]). Lanes for the two gels in each panel: L, 100 bp DNA ladder (New England Biolabs, Inc.), WDP lanes 1–14, WDP samples 1–14; SDP lanes: L, 100 bp DNA ladder, lanes 1–12, SDP samples 1–12. Samples in these gels correspond to those in Table S3, which contains the high resolution genotyping data.

Microsatellite and SNP markers were identified on either side of the candidate genes; TYR, *OCA2, TYRP1* and *SLC45A2* (Table 1 and Table S1). Because a mutation inherited from a founder dog is also linked with a region of DNA (i.e. in linkage disequilibrium, LD), a marker allele that is identified within the region of LD will always be passed along with the mutation. Additionally, an autosomal recessively inherited disease will only show the phenotype in the homozygous state, and therefore, the marker in LD with that mutation must also be homozygous for the same allele in all affected individuals. The use of markers within 500 kb on each side of the gene gives a probability of recombination between a single marker and the candidate gene to 0.005 per meiosis (assuming 1% recombination per Mb, see [14] for a full discussion). Under the assumption that the mutation in WDPs causes a recessively inherited OCA phenotype, the presence of more than one allele among the genotypes of all of the affected dogs excludes the candidate gene as being causative with a low probability of falsely excluding the true culprit gene (*p*<0.0001; http://dpca.org/BreedEd/index.php/articles/44-history/381-albinism-science). As we have previously noted, it is not necessary to genotype controls for the purpose of exclusions [14]. A candidate gene would not be excluded where homozygosity for the same marker is observed in all affected dogs and suggests the candidate may be the causative gene. It is only worth genotyping unaffected control dogs when a gene cannot be excluded to provide statistical evidence of association (analogous to the lack of a need for population allele frequency data in human paternity testing when a male is excluded as being the father because of non-sharing of alleles with the offspring, but needed where a male cannot be excluded in order to determine the probability that the genotype of the accused male matches that of the true father by chance). Markers for *TYR, OCA2,* and *TYRP1* showed more than one allele in the affected dogs and were therefore excluded as candidate genes (Figure S1). Both *SLC45A2* markers showed almost no variability in WDPs (Figure 4 A and B, left panels), but were highly variable in SDPs (Figure 4 A and B, right panels).

It is known that long microsatellites have relatively high mutation rates (>0.001 mutations/locus/generation). Therefore, single repeat differences from the most common allele are considered to be the same allele (a few of these single step mutations were observed; Table S3) [16]. The alleles for the upstream and downstream markers are fixed in the WDPs (24/24 alleles are identical for both markers). Based upon the frequency of these alleles in the SDP (11/18 alleles for the upstream marker and 0/18 alleles for the downstream marker; Table S3), this difference is highly significant (*p* = 0.0012 for the upstream markers, *p*<0.0001 for the downstream marker, Fisher’s exact test). This indicated *SLC45A2* was a candidate gene of significant interest and detailed investigation followed.

### Sequencing *SLC45A2*

The seven exons of *SLC45A2* and the nearby flanking intronic regions, as identified in the CanFam2.0 genome assembly, were sequenced in one WDP and one SDP by Sanger dideoxy sequencing. The primer set for exon 7 (the last exon for the gene) did not produce a genome browser-predicted size in the WDP sample but did produce an appropriately sized product in the SDP (data not shown). The WDP product was sequenced and matched a region on a different chromosome and we therefore concluded it was an off-target amplification product. It was hypothesized that a deletion event removed part of this exon, thereby preventing amplification of the correct target.

**Figure 5:**
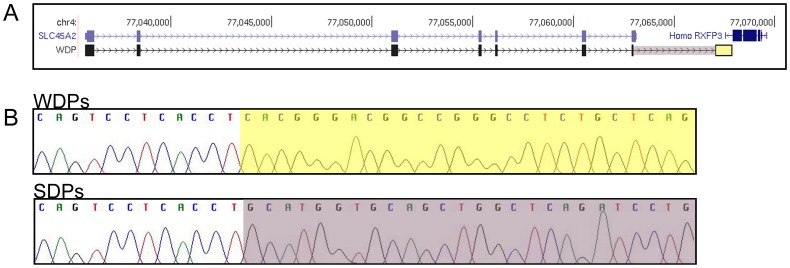
Deletion identified that includes part of exon 7 of *SLC45A2* in white Doberman pinschers. A 4,081 base pair deletion was identified in white Doberman pinschers (WDPs). The deletion occurs between chr4∶77,062,970 and chr4∶77,067,051 (UCSC Genome Browser, CanFam2.0). (A) *SLC45A2* canine exons (light blue boxes) and WDP sequenced exons (black boxes). Note that a partial deletion of exon 7 (the last exon) is seen in WDPs. The yellow box in (A) identifies the downstream break point of the WDP deletion; the WDP deletion ends before the human gene *RXFP3*. The yellow box (A) corresponds to the yellow box in (B) for WDPs. The deletion is marked with a purple box in (A) and corresponds to the beginning of the normal sequence seen in (B) for SDPs.

Identification of the deletion break points was achieved by testing a panel of short-amplicon primer sets at various distances downstream of this last exon (Figure S2). The primer set nearest to the last exon that produced a successful amplification was approximately 4 kb downstream of exon 7. Amplification across the deletion breakpoints was achieved by using a forward primer just upstream of exon 7 and the reverse primer from the nearest working downstream primer set (Table S4, Amplicon 8). The product was sequenced to identify the precise upstream and downstream deletion breakpoints. A 4,081 bp deletion was identified that included 163 bps of exon 7 and an adjacent downstream region between the base positions chr4∶77,062,968–77,067,051 (Figure 5). The WDP deletion break point ended 509 bps before the next downstream gene, *RXFP3* (chr4∶77,067,560–77,069,644). This mutation is predicted to cause the last 50 amino acids of exon 7 to be replaced by 191 new amino acids before an in-frame stop codon is found, as predicted from the canine reference genome (CanFam2.0) (Figure S3). This deletion also removes the poly-A addition signal (AATAAA), and the next predicted polyadenylation signal for the mutant chromosome is 6,106 bp downstream from the new stop codon. A genotyping assay was developed and used to test SDPs and other breeds for the deletion and the mutation was not found in homozygous form in any normal pigmented dogs but two carrier SDPs were identified (Table S5 and Figure S4).

In addition to this deletion, two SNPs and two single nucleotide variants (SNVs) were identified in the coding regions that differed between the sequenced WDPs and SDPs (Table 3). Two of these SNPs had been previously identified (rs24086317, rs9077026). Of the remaining two SNVs in the WDPs, one was a synonymous mutation (chr4∶77,051,137) while the other was nonsynonymous (chr4∶77,060,629, P250L). PolyPhen-2 predicts that this amino acid change, P250L, is benign (HumVar 0.014) [17].

**Table 3:** Single nucleotide variant locations in genomic WDP DNA.

Location	Position on Chr4^1^	Reference allele^2^	Variant allele^3^	Protein change^4^	SNP number^5^	
Exon 1	77,035,976	G	A	–	rs24086371	
Exon 3	77,051,137	C	T	–	–	
Exon 4	77,055,424	C	T	–	rs9077026	
Exon 6	77,060,629	G	A	P250L	–	

1 Gene location is based on the UCSC Genome Browser canine reference genome, CanFam2.0.

2 Reference allele from UCSC Genome Browser canine reference genome, CanFam2.0.

3 Variant allele from sequenced gDNA of WDP.

4 Protein change due to the SNV change in WDP.

5 SNP number from Broad Instituse SNP collection (http://www.broadinstitute.org/mammals/dog/snp2).

### Extent of Linkage Disequilibrium around *SLC45A2*

Microsatellite markers were designed flanking *SLC45A2* at increasing distances from the gene to identify the region linked to the mutation (Table S2). Seven Mb upstream of *SLC45A2*, 2 of 12 WDPs showed a recombination event, while 5 Mb downstream of the gene 9 of 12 WDPs showed a recombination event. These data show the region linked to the causative mutation is approximately 10 Mb (Table S3).

### cDNA Analysis and Sequencing

**Figure 6:**
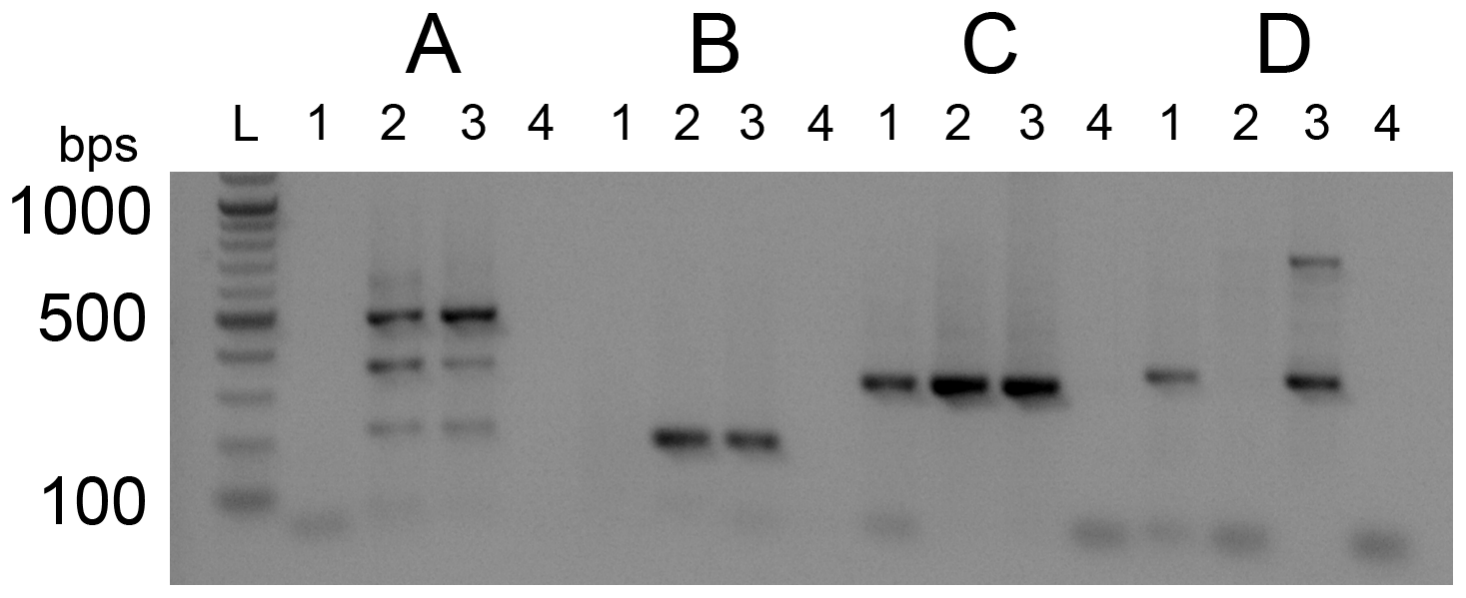
cDNA PCR-amplicons for *SLC45A2, ACTB* and *TYR* shows extremely low or absent *SLC45A2* transcript in white Doberman pinscher skin sample. cDNA was made from mRNA extracted from skin samples for lanes 1, WDP; 2, white skin from a tri-colored dog; and 3, pigmented skin from a tri-colored dog. Each lane 4 is a water blank used as a negative control. (A) *SLC45A2* product from exon 2–4, i.e. upstream of the deletion start site, (B) *SLC45A2* product from exon 6–7, i.e. bridging the deletion start site, (C) *ACTB* used for mRNA integrity control and (D) *TYR* used for mRNA integrity control as well as to show proof of concept of this assay. The two *SLC45A2* products are undetectable in the WDP sample, while the *ACTB* and *TYR* products are present with bands at similar intensity to controls. The white skin from a tri-colored dog showed undetectable *TYR* product but a similar band intensity from the pigmented skin for all other products. The L lane shows a 100 bp ladder (New England Biolabs, Ipswich, MA, USA).

In order to investigate the expression of the SLC45A2 mRNA in WDPs as compared with SDPs, RT-PCRs were performed on SLC45A2 and two control mRNAs, ACTB, and TYR. The cDNA was made from skin biopsy samples. Products consisted of exons 2–4 of *SLC45A2*, exons 6–7 of *SLC45A2*, exons 2–3 of *ACTB* and exons 2–4 of *TYR* (Table 4 and Figure 6). Of the two products within *SLC45A2,* one product was positioned upstream of the mutation site and the other product had the forward primer upstream of the mutation but the reverse primer within the deleted exonic region (Figure 6 A, B, respectively). Both products for *SLC45A2* were not detectable in WDP skin cDNA samples but were present in control pigmented skin. *ACTB* is ubiquitously expressed and was used as an RNA/cDNA quality control for the skin samples (Figure 6 C). *TYR* was predicted to have normal expression in the WDP sample and in control pigmented skin but would not be expressed in the control white skin (from a tri-colored dog) sample, as seen in Figure 6 D. Sequencing from the resultant PCR products for all targets verified that the correct cDNAs were amplified (data not shown).

**Table 4:** Primers for amplification of cDNA in a WDP and a tri-colored dog.

Amplicon ID^1^	F Primer	R Primer	Amplicon Size^2^
*SLC45A2* Exon 2/4	GCTGCTGACTTCATTGATGG	AGGCAGAGGTAATGGGAAGG	497
*SLC45A2* Exon 6/7	CCATGTTTGGTGTGATGTCC	ACCTCCAACCAGGATCTGAG	191
*ACTB* Exon 2/3	AGATCTGGCACCACACCTTC	TTCATGAGGTAGTCGGTCAGG	322
*TYR* Exon 2/4	CCAATAGGAGCATTGGCTTC	AACCATGACAAAGCCAGGAC	293

1 Amplicons were desgined across exons to ensure no contamination of genomice DNA.

2 Amplicon size as predicted by the UCSC Genome Browser canine reference genome, CanFam2.0.

## Discussion

In the first aim of this study we set out to produce a detailed description of the ocular phenotype of WDPs. Key characteristics of the WDP ocular phenotype included: photophobia and hypopigmentation of the eyelid margins, nictitating membrane margins, and cilia. All WDPs had lightly pigmented irides with transillumination defects and vertically-oriented ovoid dyscoria. Gonioscopy showed hypopigmentation of the pectinate ligament and trabecular meshwork. Indirect ophthalmoscopy revealed hypopigmentation of the retinal pigment epithelium. These findings in addition to the unique coat-color are remarkably similar to oculocutaneous albinism (OCA) observed in humans.

Interestingly, nystagmus was not a common clinical finding in the WDP examined, whereas in human patients with OCA, nystagmus is considered an important finding for clinical diagnosis [18]. The specific origin of nystagmus present in OCA patients has not been definitively localized. Two possible causes of nystagmus associated with albinism have been proposed: (1) misrouting of fibers within the optic nerve and optic tract due to an unknown pathophysiological mechanism, and (2) abnormal visual function and acuity caused by the foveal hypoplasia resulting from decreased or absent macular pigmentation [18], [19].

Nystagmus is also not observed in dogs with *TYRP1* mutations. Although we included *TYRP1* as a formal candidate gene, the only apparent impact of *TYRP1* mutations in dogs is the production of a brown coat and planum nasale [1]. This may suggest that optic nerve fibers are not misrouted or are misrouted to a lesser degree in canine OCA compared with humans and perhaps other mammalian species [18]. Although the dog does have a cone photoreceptor-enriched retinal region called the visual streak/area centralis, this area is not directly analogous to the human macula and fovea. The lack of a fovea in the dog and presumably any associated albinism-induced foveal hypoplasia may also contribute to a lack of nystagmus observed in this study. Finally, the pattern of nystagmus has been observed to change with advancing age in humans with OCA from a high amplitude, low frequency pattern in infancy to a typical jerk pattern in adults with an average frequency of 3.3 Hz [20]. As the WDPs in this study were collected into large groups before examination, which produced a certain level of underlying excitement among all of the dogs, we cannot exclude the possibility a subtle low-frequency nystagmus was simply undetected. Further investigation is required to determine if nystagmus is indeed present in the WDP and ultimately what anatomical abnormality produces the nystagmus. The WDP will likely be an excellent model to perform in vivo structure/function studies utilizing OCT imaging coupled with ERG and VEP testing to investigate the potential origin of nystagmus in human OCA patients [21], [22].

For the second aim we set out to objectively determine if an increased prevalence of ocular and cutaneous melanocytic tumors was present in WDP. Our original intent was to count the number of tumors and nevi on each WDP. However, the large number and small size of some of the tumors and nevi made it impractical to obtain accurate lesion counts under the time constraints in which we had access to the dogs. Anecdotal information provided to the authors by WDP owners indicate that WDPs are born blemish free and acquire the nevi and masses over time. The number of WDPs with masses was significantly higher than in SDPs (p<0.0001), and the presence of dermal masses appeared to increase with age. These findings suggest that the albinism phenotype and/or the underlying mutation in *SLC45A2* are risk factors for development of cutaneous and ocular masses.

Of particular interest, in 11 cutaneous masses excised from 8 different WDPs, all showed proliferation of melanocytic cells with varying histologic indicators of malignancy. If the significant reduction in skin pigmentation alone, resulting in damage of cutaneous tissue from environmental ultraviolet radiation, were to blame for the increased frequency of masses detected in WDPs, a range of neoplastic cell-types such as squamous cell carcinoma and basal cell carcinoma might be expected in addition to the melanocytic lesions observed in this study. Skin tumors found in humans with OCA are most frequently squamous cell carcinoma, followed by basal cell carcinoma, and, more rarely, melanoma [23]–[25]. The proportion of these tumor types in human albinism seems to be very similar regardless of the locus involved, although the frequency of skin tumors compared with non-albinos is considerably greater [23]–[25]. As the number of dogs from which skin tumors were obtained in this study was low, we cannot rule out the possibility of some form of bias in the choice of masses selected for biopsy that resulted in the overwhelming number of melanocytomas and melanomas diagnosed histologically. However, this finding may also lead to the intriguing hypothesis that the partial deletion of exon 7 of *SLC45A2* and resultant absence of protein expression or abnormal protein function may specifically produce an environment within melanocytes that significantly contributes to the risk for neoplastic and/or malignant transformation. In humans, a nonpathogenic SNP located in an intronic region of *SLC45A2* has been identified in which the major allele is significantly associated with malignant melanoma and the minor allele is protective [26]–[29]. Additionally, nonpathogenic variants in *SLC45A2* have been associated with the development of lentigines on Japanese women’s cheeks and German women’s arms [29]. There is some evidence that melanomas are not strictly resultant from UV exposure, but are caused by something inherent to the melanosome, particularly in melanocytes with mutations in OCA and other light-skin associated genes [30]–[32]. The difference in tumor type in WDPs could be attributable to a true species difference in SLC45A2 function(s), or perhaps due to the effect of a linked variant among the approximately 44 genes in the 10 Mb region of homozygosity surrounding the mutant gene (Table S6).

For the third aim we screened DNA samples from WDPs to determine if a genetic mutation in any of the genes known to cause human OCA resulted in the “white” phenotype. Here we have described a partial deletion of the last exon of *SLC45A2* that is associated with the white Doberman pinscher phenotype (*p*<0.0001). The *TYR* gene had previously been excluded as containing any coding variation in WDPs [33]. Although mutations affecting the regulatory elements of a recessively inherited phenotype are rare, we formally examined this possibility for *TYR* (as well as other candidate genes) using exclusion analysis; *TYR* was excluded as well as *OCA2* and *TYRP1*. We were aware of only these four OCA loci at the time the work was started, but exclusion analysis for the OCA5-7 loci would have been carried out if the mutation in *SLC45A2* had not been identified.

Exclusion analysis is a method for quickly eliminating a majority of candidate genes by utilizing variable markers (SNPs and MS) so that more time and resources can be spent on genes that are not excluded. In relatively common recessive diseases in founder populations, all affected animals are homozygous for the same mutant allele. Similarly, for markers in complete linkage disequilibrium with the mutation, the affected dogs must also have two copies of the same marker allele. The presence of non-shared marker alleles in affected dogs excludes that candidate gene as being the possible causative gene.

*SLC45A2* is a putative sugar transporter, showing sequence identity to sugar transporters in plants and to a H^+^/sucrose symporter in *Drosophila* [34]. Evidence suggests that the protein causes melanosome acidification, which is necessary for protein sorting and enzymatic activity during melanogenesis [35]. Sequence variations in the promoter region of *SLC45A2* are associated with the historical variation in normal skin pigment between human populations living in northern versus southern regions of the world [36]. In a population of over 1,000 Caucasians, a 3 base pair duplication and a SNP (c. -1176_-1174dupAAT and c.-1169G>A, respectively) were found to have higher allele frequencies in individuals with olive skin compared with those that had fair skin.

Pathogenic mutations in *SLC45A2* are responsible for OCA4 in humans and “white” (actually pale cream) phenotypes described in Bengal tigers, horses, Western lowland gorilla and mice [37]–[41]. OCA4 is the third most common form of OCA worldwide, and is also one of the most common forms of OCA in Japan [42]. OCA4 patients present with varying degrees of hypopigmentation of the skin and hair as well as visual abnormalities. The variation is due to the severity of the mutation; some missense and frame-shift mutations result in low or no pigmentation in skin, hair or eyes and nystagmus while some nonsynonymous mutations allow the production of some pigment [40], [42], [43]. All pathogenic mutations are located in the transmembrane regions, except one, a splice site variation that causes the skipping of exon 2 and loss of a transmembrane region, indicating the importance of sequence conservation in these domains [42], [43].

The gene *SLC45A2* was examined for a mutation that would disrupt its function. A 4,081 bp deletion was identified, including the last 163 bps of the last exon of *SLC45A2* (Figure 5). The downstream breakpoint is 509 bp from the 3′ end of *RXFP3*, which is transcribed in the opposite direction compared with *SLC45A2*. If expressed, the mutant protein is predicted to lose wild-type amino acids after position 481, which are replaced by 191 amino acids that have no sequence identity with the wild-type protein (Figure S3). Four additional variations were identified in the genomic sequence of WDPs but these variations are not predicted to be causative of the OCA phenotype.

Examination of the *SLC45A2* mRNA in WDP skin indicated a reduced amount of this mRNA compared with pigmented skin from a control dog. Degradation of the mRNA might not be expected by the known nonsense-mediated mRNA decay mechanism because an open reading frame is maintained into the final exon before a termination codon is encountered [44]. A more likely reason for the reduced amount of mRNA is based upon the long 3′UTR that is predicted to result from the deletion. The deletion removes the wild-type poly-A addition signal (AATAAA) normally associated with *SLC45A2.* The next signal that is available for use is predicted to be 6,106 bp downstream of the new stop codon. It has been shown that mRNAs with 3′ untranslated regions longer than 3 kb tend to be unstable and are degraded, possibly through binding of Upf1 [45].

If a small amount of mutant protein is actually produced from the mutant transcript, it is predicted that the protein would not be functional. Missense mutations in exon 7 in other species are associated with light colored or albino phenotypes [37], [39], [42], [43], [46]. Additionally, the loss of the last 50 amino acids includes almost two entire transmembrane domains and replaces these amino acids with 191 new amino acids (http://www.uniprot.org/uniprot/Q9UMX9). These observations, combined with the known association of OCA4 with mutations in *SLC45A2* in albino individuals of other species, lead us to the conclusion that the identified deletion causes OCA4 in white Doberman pinschers. Further work is needed to distinguish among the various possibilities for the increased prevalence of melanocytic neoplasms and the WDP presents a valuable large animal model for this work. The WDP should also serve as a valuable model for OCA4 vision disturbances and possible gene therapy for the reduction of visual defects.

## Supporting Information

Figure S1 **Exclusion of the candidate genes** ***TYR*** **,** ***OCA2*** **and** ***TYRP1*** **as the culprit gene.** 2.0% agarose gels of microsatellite (MS) or single nucleotide polymorphism (SNP) markers with white Doberman pinscher DNA. (A) *TYR* MS-1, (B) *TYR* MS-2, (C) *OCA2* SNP-1, (D) *OCA2* SNP-2, (E) *TYRP1* SNP-1 and (F) *TYRP1* MS-1. These three candidate genes were excluded by both markers tested due the lack of homozygosity for a shared single allele seen in affected dogs. Lanes in panels A–F are: L, DNA ladder (100 bp ladder, New England Biolabs, Inc.), 1–14, WDP 1–14. Marker details are contained in Table 1.
(DOCX)

Figure S2 **Identification of the boundaries of the WDP deletion.** Initial amplification using Primer set 1 (Primer1 in Table S4) suggested that exon 7 is the location of the mutation in WDPs (not shown). The reverse primer for set 1 is in the deleted region (above) and only a product of an incorrect size was amplified in WDP samples and was shown to be an off-target amplification product by sequencing. Exon 7 in SDPs, on the other hand, amplified and sequenced as expected. Subsequent primer pairs (2 and 3) downstream of exon 7 amplified in SDPs but not in WDPs. Starting much further downstream (approximately 16,200 bp downstream of F Primer1), primer sets were designed decreasing distances from exon 7 (i.e. back towards *SLC45A2*). Primer set 8 was the amplicon closest to exon 7 that successfully amplified. Finally, using the F Primer1 and R Primer8, a product was amplified and the deletion breakpoints were identified. Primer sequences and amplicon sizes are listed in Table S4.
(DOCX)

Figure S3 **SLC45A2 amino acid alignments.** Sequence alignments performed using muscle alignment in SeaView software [47]. The WDP additional amino acids are highlighted in yellow. The amino acid change by the SNV in the WDPs is highlighted in red. * indicate amino acids conserved across all species, : indicate amino acids conserved across all species, excluding WDPs. The red arrow indicates the location of the albino tiger mutation (A477V) [37]. Teal highlight indicates the albino gorilla (G518R) mutation [39]. Purple highlight indicates the cream coat color in horses mutation (N153D) (also described in humans and mice) [38]. Green highlight indicates missense mutations underlying OCA4 in humans [43].
(DOCX)

Figure S4 **WDP mutation genotyping assay.** PCR based and agarose gel electrophoresis diagnostic assay for identifying the presence or absence of the WDP deletion.
(DOCX)

Table S1 **Primers for markers used in exclusion analysis.**
(XLSX)

Table S2 **Microsatellite marker information for linkage disequilibrium surrounding SLC45A2.**
(XLSX)

Table S3 **Extent of linkage disequilibrium surrounding SLC45A2.**
(XLSX)

Table S4 **Primers used to locate the approximate position of the deletion.**
(XLSX)

Table S5 **Breeds tested for SLC45A2 deletion.**
(XLSX)

Table S6 **List of genes within the ∼10 Mb region of homozygosity.**
(XLSX)
