# Supplementary material for: A Partial Gene Deletion of SLC45A2 Causes Oculocutaneous Albinism in Doberman Pinscher Dogs
Source: PLoS One. 2014 Mar 19;9(3):e92127. doi: 10.1371/journal.pone.0092127 (PMC3960214; doi:10.1371/journal.pone.0092127)
Supplement: Figure S1 — Exclusion of the candidate genes TYR , OCA2 and TYRP1 as the culprit gene. 2.0% agarose gels of microsatellite (MS) or single nucleotide polymorphism (SNP) markers with white Doberman pinscher DNA. (A) TYR MS-1, (B) TYR MS-2, (C) OCA2 SNP-1, (D) OCA2 SNP-2, (E) TYRP1 SNP-1 and (F) TYRP1 MS-1. These three candidate genes were excluded by both markers tested due the lack of homozygosity for a shared single allele seen in affected dogs. Lanes in panels A–F are: L, DNA ladder (100 bp ladder, New England Biolabs, Inc.), 1–14, WDP 1–14. Marker details are contained in Table 1. (DOCX) [file pone.0092127.s001.docx]

**
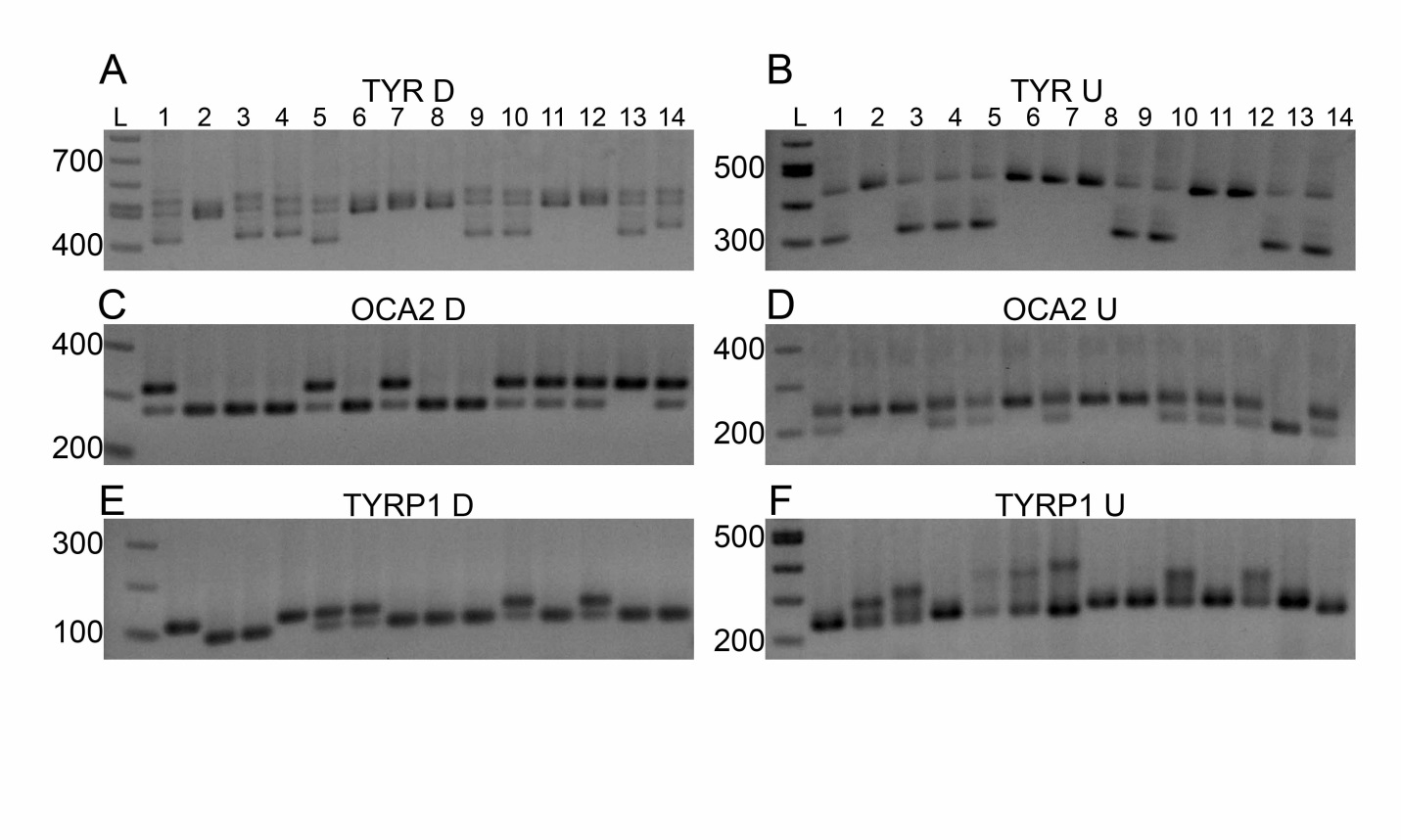
Supplemental Figure 1. Exclusion of the candidate genes *TYR*, *OCA2* and *TYRP1* as the culprit gene.**

2.0% agarose gels of microsatellite (MS) or single nucleotide polymorphism (SNP) markers with white Doberman pinscher DNA. (A) *TYR* MS-1, (B) *TYR* MS-2, (C) *OCA2* SNP-1, (D) *OCA2* SNP-2, (E) *TYRP1* SNP-1 and (F) *TYRP1* MS-1. These three candidate genes were excluded by both markers tested due the lack of homozygosity for a shared single allele seen in affected dogs. Lanes in panels A-F are: L, DNA ladder (100 bp ladder, New England Biolabs, Inc.), 1-14, WDP 1-14. Marker details are contained in Table 1.
