# Supplementary material for: A Partial Gene Deletion of SLC45A2 Causes Oculocutaneous Albinism in Doberman Pinscher Dogs
Source: PLoS One. 2014 Mar 19;9(3):e92127. doi: 10.1371/journal.pone.0092127 (PMC3960214; doi:10.1371/journal.pone.0092127)
Supplement: Figure S2 — Identification of the boundaries of the WDP deletion. Initial amplification using Primer set 1 (Primer1 in Table S4) suggested that exon 7 is the location of the mutation in WDPs (not shown). The reverse primer for set 1 is in the deleted region (above) and only a product of an incorrect size was amplified in WDP samples and was shown to be an off-target amplification product by sequencing. Exon 7 in SDPs, on the other hand, amplified and sequenced as expected. Subsequent primer pairs (2 and 3) downstream of exon 7 amplified in SDPs but not in WDPs. Starting much further downstream (approximately 16,200 bp downstream of F Primer1), primer sets were designed decreasing distances from exon 7 (i.e. back towards SLC45A2). Primer set 8 was the amplicon closest to exon 7 that successfully amplified. Finally, using the F Primer1 and R Primer8, a product was amplified and the deletion breakpoints were identified. Primer sequences and amplicon sizes are listed in Table S4. (DOCX) [file pone.0092127.s002.docx]

**Supplemental Figure 2. Identifying the boundaries of the WDP deletion**

Initial amplification using Amplicon 1 (Amplicon1 in Table S4) suggested that exon 7 is the location of the mutation in WDPs (not shown). The reverse primer for set 1 is in the deleted region (above) and only a product of an incorrect size was amplified in WDP samples and was shown to be an off-target amplification product by sequencing. Exon 7 in SDPs, on the other hand, amplified and sequenced as expected. Subsequent amplicons (2 and 3) downstream of exon 7 amplified in SDPs but not in WDPs. Starting much further downstream (~16,200 downstream of Amplicon1), primer sets were designed decreasing distances from exon 7 (i.e. back towards *SLC45A2*). Amplicon 8 was the closest to exon 7 that successfully amplified. Finally, using the F primer from Amplicon1 and R primer from Amplicon8, a product was amplified and the deletion breakpoints were identified. Primer sequences and amplicon sizes are listed in Table S4.

**
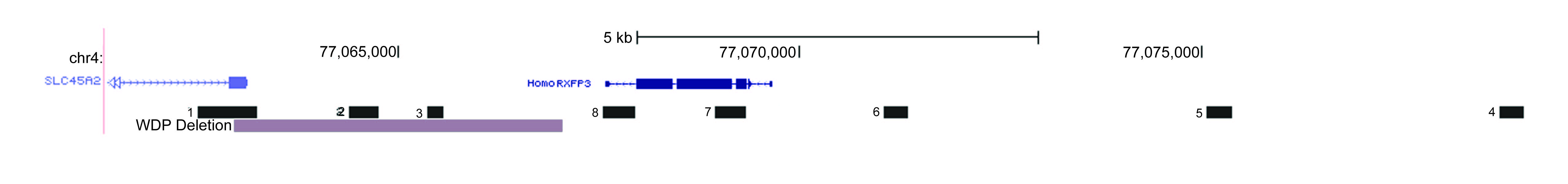
**
