# Supplementary material for: A Partial Gene Deletion of SLC45A2 Causes Oculocutaneous Albinism in Doberman Pinscher Dogs
Source: PLoS One. 2014 Mar 19;9(3):e92127. doi: 10.1371/journal.pone.0092127 (PMC3960214; doi:10.1371/journal.pone.0092127)
Supplement: Figure S3 — SLC45A2 amino acid alignments. Sequence alignments performed using muscle alignment in SeaView software [47]. The WDP additional amino acids are highlighted in yellow. The amino acid change by the SNV in the WDPs is highlighted in red. * indicate amino acids conserved across all species, : indicate amino acids conserved across all species, excluding WDPs. The red arrow indicates the location of the albino tiger mutation (A477V) [37]. Teal highlight indicates the albino gorilla (G518R) mutation [39]. Purple highlight indicates the cream coat color in horses mutation (N153D) (also described in humans and mice) [38]. Green highlight indicates missense mutations underlying OCA4 in humans [43]. (DOCX) [file pone.0092127.s003.docx]

**Supplemental Figure 3. SLC45A2 amino acid alignments.**

Sequence alignments performed using muscle alignment in SeaView software [47]. The WDP additional amino acids are highlighted in yellow. The amino acid change by the SNV in the WDPs is highlighted in red. * indicate amino acids conserved across all species, : indicate amino acids conserved across all species, excluding WDPs. The red arrow indicates the location of the albino tiger mutation (A477V) [37].Teal highlight indicates the albino gorilla (G518R) mutation [39]. Purple highlight indicates the cream coat color in horses mutation (N153D) (also described in humans and mice) [38]. Green highlight indicates missense mutations underlying OCA4 in humans [43].

1

[WhiteDoberman] MGGNTGQLGI HTCKSLAEDD HFDPVEPPKR PTSNLIMHSM AMLGREFCYA VEAAYVTPVL

[Canine] MGGNTGQLGI HTCKSLAEDD HFDPVEPPKR PTSNLIMHSM AMLGREFCYA VEAAYVTPVL

[Human] MGSNSGQAGR HIYKSLADDG PFDSVEPPKR PTSRLIMHSM AMFGREFCYA VEAAYVTPVL

[Mouse] MSGSNGPTDT HTYQSLAEDC PFGSVEQPKR STGRLVMHSM AMFGREFCYA VEAAYVTPVL

[Horse] MGGNSGQPGV PTYKSLAEDG PFGSVELPKR STGRLVMHSM AMFGREFCYA VEAAYVTPVL

[Gorilla] MGGNGGQAGR HIYESLAHDG PFDSVEPPKR PTSRLIMHSL AMFGREFCYA VEAAYVTPVL

* * *** * * ** *** * * *** ** ******* **********

61

[WhiteDoberman] LSVGLPKSLY STVWLLSPIL GFLLQPVGGS ASDNCQARWG RRRPYILTLG IMMLLGMALY

[Canine] LSVGLPKSLY STVWLLSPIL GFLLQPVGGS ASDNCQARWG RRRPYILTLG IMMLLGMALY

[Human] LSVGLPSSLY SIVWFLSPIL GFLLQPVVGS ASDHCRSRWG RRRPYILTLG VMMLVGMALY

[Mouse] LSVGLPKSLY SMVWLLSPIL GFLLQPVVGS ASDHCRARWG RRRPYILTLA IMMLLGMALY

[Horse] LSVGLPKRLY SVVWLLSPVL GFLLQPVVGS ASDHCRARWG RRRPYILALS VIMLLGMALY

[Gorilla] LSVGLPSSLY SIVWFLSPIL GFLLQPVVGS ASDHCRSRWG RRRPYILTLG VMMLVGMALY

***** ** * ** ***** ******* ** *** * *** ******* * ** *****

121

[WhiteDoberman] LNGDAVVSAL IADPRRKRIW AITITMIGVV FFDFAADFID GPIKAYLFDV CSYEDKERGL

[Canine] LNGDAVVSAL IADPRRKRIW AITITMIGVV FFDFAADFID GPIKAYLFDV CSYEDKERGL

[Human] LNGATVVAAL IANPRRKLVW AISVTMIGVV LFDFAADFID GPIKAYLFDV CSHQDKEKGL

[Mouse] LNGDAVVSAL VANPRQKLIW AISITMVGVV LFDFSADFID GPIKAYLFDV CSHQDKEKGL

[Horse] LNGDAVISAL IADRRKKLTW AITITMIGVV LFDFAADFID GPIKAYLFDV CSHQDKERGL

[Gorilla] LNGATVVAAL IANPRRKLVW AISVTMIGVV LFDFAADFID GPIKAYLFDV CSHQDKEKGL

*** * ** * * * * ** ** *** *** ***** ********** ** *** **

181

[WhiteDoberman] HYHAFFTGFG GALGYLLGAI DWAHLEIGRV LGSEFQVMFF FSALVLTLCF IIHLCSIPEA

[Canine] HYHAFFTGFG GALGYLLGAI DWAHLEIGRV LGSEFQVMFF FSALVLTLCF IIHLCSIPEA

[Human] HYHALFTGFG GALGYLLGAI DWAHLELGRL LGTEFQVMFF FSALVLTLCF TVHLCSISEA

[Mouse] HYHALFTGFG GALGYILGAI DWVHLDLGRL LGTEFQVMFF FSALVLILCF ITHLCSIPEA

[Horse] HHHALFTGLG GALGYILGAI DWAHLKLGRM LGTEFQVMFF FSALMLTLCV VIHLCSIPEA

[Gorilla] HYHALFTGFG GALGYLLGAI DWAHLELGRL LGTEFQVMFF FSALVLTLCF IVHLCSISEA

* ** *** * ***** **** ** ** ** ** ******* **** * ** ***** **

241

[WhiteDoberman] PLRDVTKDIL PQQAPQDFLL SSDKMYQYGS IEKAKNGYVN PELALQGEKT PNPSKQISKT

[Canine] PLRDVTKDIP PQQAPQDFLL SSDKMYQYGS IEKAKNGYVN PELALQGEKT PNPSKQISKT

[Human] PLTEVAKGIP PQQTPQDPPL SSDGMYEYGS IEKVKNGYVN PELAMQGAKN KNHAEQTRRA

[Mouse] PLRDAATDPP SQQDPQGSSL SASGMHEYGS IEKVKNGGAD TEQPVQEWKN KKPSGQSQRT

[Horse] PLRDVAKDIP PQQDSQDPLL SSDRMYEYGS IEKVKNGYIN PEMVLQGEKT TN-TQQTRRT

[Gorilla] PLTDVAKGIP PQQTPQDPPL SSDGMYEYGS IEKVKNGYVN PELAMQGAKN KNHAEQTRRA

** : ** * * * * *** *** *** * * * *

301

[WhiteDoberman] MTMTSLLRVL MNMPSHYLCL CISHFIGWTA FLSNMLFFTD FMGQIVYHGD PYSAHNSTEF

[Canine] MTMTSLLRVL MNMPSHYLCL CISHFIGWTA FLSNMLFFTD FMGQIVYHGD PYSAHNSTEF

[Human] MTLKSLLRAL VNMPPHYRYL CISHLIGWTA FLSNMLFFTD FMGQIVYRGD PYSAHNSTEF

[Mouse] MSMKSLLRAL VNMPSHYRCL CVSHLIGWTA FLSNMLFFTD FMGQIVYHGD PYGAHNSTEF

[Horse] MTMKSLLRAL VSMPPHYRYL CISHLLGWTA FLSNMLFFTD FMGQIVYHGD PYSAHNSTEF

[Gorilla] MTLKSLLRAL VSMPPHYRYL CISHLIGWTA FLSNMLFFTD FMGQIVYRGD PYSAHNSTEF

* **** * ** ** * * ** **** ********** ******* ** ** *******

361

[WhiteDoberman] LIYERGVEVG CWGLCINSVF SSLYSYFQKP LVSYIGLKGL YFTGYLLFGL GTGFIGLFPN

[Canine] LIYERGVEVG CWGLCINSVF SSLYSYFQKP LVSYIGLKGL YFTGYLLFGL GTGFIGLFPN

[Human] LIYERGVEVG CWGFCINSVF SSLYSYFQKV LVSYIGLKGL YFTGYLLFGL GTGFIGLFPN

[Mouse] LIYERGVEVG CWGLCINSVF SSVYSYFQKA MVSYIGLKGL YFMGYLLFGL GTGFIGLFPN

[Horse] LIYQRGVEVG CWGLCINSVF SSLYSYFQKV LVSYVGLKGL YFMGYLLFGL GTGFIGLFPN

[Gorilla] LIYERGVEVG CWGLCINSVF SSLYSYFQKV LVSYIGLKGL YFTGYLLFGL GTGFIGLFPN

*** ****** *** ****** ** ****** *** ***** ** ******* **********

421 ↓

[WhiteDoberman] VYSTLALCTM FGVMSSTLYT VPFNLIAKYH REE-QEKRQQ ARGGSLDSGE RGQGLDCAVL

[Canine] VYSTLALCTM FGVMSSTLYT VPFNLIAKYH REE-QEKRQQ ARGGSLDSGE RGQGLDCAVL

[Human] VYSTLVLCSL FGVMSSTLYT VPFNLITEYH REE-EKERQQ APGGDPDNSV RGKGMDCATL

[Mouse] VYSTLVLCSM FGVMSSTLYT VPFNLIAEYH REEEKEKGQE APGG-PDNQG RGKGVDCAAL

[Horse] IYSTLVLCTS FGVMSSTLYT VPFNLIAEYH REEQEKQRRQ AQGGDVDSSG RGQGLDCAAL

[Gorilla] VYSTLVLCSL FGVMSSTLYT VPFNLITEYH REE-EKERQQ APGGDPDNSV RGKGMDCATL

**** ** ********** ****** ** *** * ** ** * *** *

481

[WhiteDoberman] T--------- ---------- ---------- ---------- ---------- -SRDGRASAQ

[Canine] TCMVQLAQIL VGGGLGFLVN KAGSV-IVVI TASALALIGC CFVALFVRYV D---------

[Human] TCMVQLAQIL VGGGLGFLVN TAGTVVVVVI TASAVALIGC CFVALFVRYV D---------

[Mouse] TCMVQLAQIL VGGGLGFLVN MAGSVVVVVI TASAVSLIGC CFVALFVRYV D---------

[Horse] TCMVQLAQIL VGGGLGFLVN IAGSVVVVVI TASVVALIGC CFVVLFVRYV A---------

[Gorilla] TCMVQLAQIL VGGGLGFLVN TAGTVVIVVI TASAVALIGC CFVALFVRYV D---------

*::::::::: :::::::::: :: : ::: ::: : :::: ::: ::::::

541

[WhiteDoberman] SPTGHVSCPF PFSGRGLRRS RFSPPLAAAA PSRSDVIASA AWRPAFCLGG HLSPGSRVHT

[Canine] ---------- ---------- ---------- ---------- ---------- ----------

[Human] ---------- ---------- ---------- ---------- ---------- ----------

[Mouse] ---------- ---------- ---------- ---------- ---------- ----------

[Horse] ---------- ---------- ---------- ---------- ---------- ----------

[Gorilla] ---------- ---------- ---------- ---------- ---------- ----------

601

[WhiteDoberman] LARGAINGRW PRGRCLSRCL SLAGGARSSP LSSSSGEVSE PGPRAATQRG LPRQGAKGGF

[Canine] ---------- ---------- ---------- ---------- ---------- ----------

[Human] ---------- ---------- ---------- ---------- ---------- ----------

[Mouse] ---------- ---------- ---------- ---------- ---------- ----------

[Horse] ---------- ---------- ---------- ---------- ---------- ----------

[Gorilla] ---------- ---------- ---------- ---------- ---------- ----------

661

[WhiteDoberman] RRRPPPSWVR LEDSLRSSRD VSAMHAPNCP RTTWLSPASS RPSFPTFRIL FSASGHPDPA

[Canine] ---------- ---------- ---------- ---------- ---------- ----------

[Human] ---------- ---------- ---------- ---------- ---------- ----------

[Mouse] ---------- ---------- ---------- ---------- ---------- ----------

[Horse] ---------- ---------- ---------- ---------- ---------- ----------

[Gorilla] ---------- ---------- ---------- ---------- ---------- ----------

721

[WhiteDoberman] QG

[Canine] --

[Human] --

[Mouse] --

[Horse] --

[Gorilla] --
