# Supplementary material for: A Partial Gene Deletion of SLC45A2 Causes Oculocutaneous Albinism in Doberman Pinscher Dogs
Source: PLoS One. 2014 Mar 19;9(3):e92127. doi: 10.1371/journal.pone.0092127 (PMC3960214; doi:10.1371/journal.pone.0092127)
Supplement: Figure S4 — WDP mutation genotyping assay. PCR based and agarose gel electrophoresis diagnostic assay for identifying the presence or absence of the WDP deletion. (DOCX) [file pone.0092127.s004.docx]

**Supplemental Figure 4. WDP mutation genotyping assay.**

PCR based and agarose gel electrophoresis diagnostic assay for identifying the presence or absence of the WDP deletion. Above, WDPs (1,2 and 3), SDP (4), mixed breed (5) and dachshund (6). M, 100 bp ladder (NEB, cite). Below, pigmented dog sequence (CanFam2.0). Green highlighted, Exon 7; blue text, WDP sequence; black text, pigmented dog sequence (consequently the deletion in WDPs); red text, primer sequences.

F_Primer_A and R_Primer_B

745 in SDPs (won’t work in WDP because R_Primer_B is within the deleted region)

F_Primer_A and R_Primer_C

495 bps in WDP, 4,576 bps in SDP/pigmented dog (not seen)

CAGTTTCTTGGTGACTGTAAAGC(F_Primer_A)AAGGACTGGGCAAACTACATCTCATGGGCCAAATGTGGCCCGCCGCCTGCTTTCGTACGGCTGGTGAGCTAAGAATTCTCTTAAAATTTTTAAATGCTATGTTACAAGAAAATCAATAGAAGGGTAACATTTGTGACAAGTGAAAATGATATGAAATCCAAATACCAATGTCCATTAGGTCATATTGGAACACTGACTCGTTTATCCACTGCCGCCGCTTTTACACCACAATGGCAGCGTTGTGTGGTTGCAACCAAGACCATGAAAGGATTTCCTACCTGGTTCTTCACAGAAAAAGTGTGCCAGCTTCTGCCCCAATGGACTGTTCATGTAGATTAAATGTCTTCCTGTGTTTCTCTTGCAGAGACAGCAGGCCCGAGGAGGGAGCCTGGACAGCGGCGAGAGAGGGCAGGGCCTGGACTGCGCAGTCCTCACCTGCATGGTGCAGCTGGCTCAGATCCTGGTTGGAGGTGGCCTGGGCTTTCTGGTCAACAAGGCCGGGAGCGTCATCGTGGTCATCACGGCCTCTGCGCTGGCGCTGATCGGCTGCTGTTTTGTGGCTCTCTTTGTTAGATACGTGGATTAGGTCAATAAAGAGACACTGTCCCTACTCTCAGACACAGGGAAGTACATGGCAGGAGATCAGTCTCTTCTTTTGGCTCTGGAGTTTTGGGGTCTGCCCACTGGACACCCTGTGTTATGACTTCAGCCTCAACAATCTG(R_Primer_B)CCCACACCTCACTCCTCTGCACAACAGTTTGCTGCTGTCAGGCATCGCTTATCTTAGGAGAGGCTGGGCTCTGCCTCCATTCAGTGGGGCAAAGGCTGAGTGTGGGAGAGAGCGCCTACCTCTCCTGGTATTTCCAGCCTTGAGAGAACTCGAGAAGCAGACTGGTTGTCCACATGACAACTGAGTTTATTCTATCATGTGTTTTCTTTTTCTTAATCAGAGTTCCGTATTTTGTTGGAGGGTCATATTTTTCACTAATTCACCAATACTCAAAATATTACTGATGATTTATTTTACCATATTAATTTACTGGTATTTAATTCATATTTGATTTCCTCATATAGACAGTCCCTGACTTAACAACAGTTCGACTTACCAAATTTTTTTTTTTTTTTTTTACTTTATGACACTTGGAAAGCAATGCACATTCAGTAGAAACCCATACGTCAAATTTTGAATTTTGATCTTTTCTAGATGGTATGTGGTATGATCCTTTCTCGTGATGCTAGGCAGTGGCAGCCACAGCTCCCTGTCAGCCTCACAATCATAAAGGTGGACAACTGGTACATTGATGACCATTCTGTCCCCATACAGCTGTGTTGTCTTTTTCATGTTCAGTGTAGTATTCAATAAATTACATGAGATAGTCAATACTCAAAACCACTCTATCTTCCCCAGTCTTCCTTCCTCCCTCTCCGTCACATTATCTGTCCTAGAGCTGCAAGGTCATGACTGAGTTAGGGGAACCATCAAGGACACCACCTGGTCCCTTCCCCATAAGTGTGGGATAATGATGAGCAAATGCTCAGAGGACCCCATGTGACTCAATACCCTGCACTATAAAAGAAATGGTACTGAGCCACCTCTCTTGCTTTTCCCATCTGTTCCTGACCTCTGTAAGGTTTAAAAATGGAATTATTTGCAGAGTTCAATGTTTATTTCCTGATCATTTCTCCAAAGACTGTTCTGGTGATTGAAGATTTTGAAATCAGTGAATCAGGTGAGCTAGGAGGGTGACCTGCAGCTGAGGACCATCTTACCTGGACCCCAAGAAGTTAAAAGTAGTCATCTCTGGCCAACCACAGGGCATGGTCTTTATTACAAGTGAAGCTCTTTTCAGCAGGAGCACTGGGTGGGGCGGCTGGCACGAAGGTAGGAGCGAGAGCATGCATCCACCACCACCCCCATCCTCCTTATATACACACTCACACACACTCAAATATTCAAATAAACACTCTGTTCTTTTAACTCAACTTTACTGAGGTTTGAATGAGTGGATAGAAGCCAACTTCAAAGGCTTTTAACCACCTTTGAGAACTCAGTATACTGAGTATACTTGTTATCAGTATACCTATGATATGTTTCCTCTCTGGGACAAAAATGTTCCATTCCCAGACAAATAAAACAAATACAGGGACACCTAAATTATGAACAGAAATCCCAGGAAATTCTGGGAAAGAAAAGGAAGCCAAACTGCTTTGCTTCCTTCTTCCCCAACCATGCAGCTGACATTCCAATCTCCAACCTTTAGTCACTGCTTTTCCTCTGCTTCAAATGTCCTCTCTATGGCCCTACGTGAGGACCCTGCCTGTTTAAATTGCCATTCCTGCCCATCTACATTGTACCCATATTTCTAGGTCCATCTCAAATACTACTTTGATGAGGAAGTCTCTGACTTCCCAGCAGAGCCAAGTTTTCCATCCCCTATATTCCTTTTTTTTTTTTAATAATTTTTTTTTAAATTTATTTATGATAGTCACAGAGAGAGAGAGAGAGAGAGAGAGAGAGAGGCAGAGACACAGGCAGAGGGAGAAGCAGGCTCCATGCACCGGGAGCCTGACGTGGGATTCGATCCTGGGTCTCCAGGATCGCGCCCTGGGCCAAAGGCAGGCGCCAAACCGCTGCGCCACCCAGGGATCCCTATCCCCTATATTCCAATAGCCCTTTCTTTTAGCCCTTACTATGACTTTCAACTCCTTGTTCCATGGCTTATTGACCTCGTGTGCGTGGCTGTGTCTCCTTTTAAATGTAAAAGTAACGTATCTAGTTGATATATGTCAGCTCAGCACCCAGTACAGGGCTTGGCACACAACTGGCATCCAAAAAATAAAATACATTTGGGCCATTTCCTAGTTTATTTTTTTCTCTTTGTAGTAGACAGAAGTCATAGTCTTTGCTCCCCCCAGCCAGCTTCTCAGAAGCCCTCAGGTGCAGAGGGGACCAGTTTGTTCTTTTTGCAGTGTTCTTTCACGAAGGCCGTGTCCCTAGCCCCTCCTGTGTTTCTGTTACCCATCACAGCAGAAGCACACAGTTCAACATACTTTCCTAAAAACCCCAAGCACAGAATTTTATTGTCAAAATTGAAATGGCAAGGTCCTTTAATAAAGAGAGTCTCTCAGTATGAGTCTAATATCAGCATCATACATGGATACACTACCATGCTACTACTGTTTGTTCTTTCTGGGTGTTCACGTGCATGTTTATTTTGTTTGGGGGGACTTGGAGGGGCTTGCGGTTCAATTTTTGTTAGCCCAATTGCTACTCAATATCAGTATCAATAATTTTTCTTCCTAGAGGGCAAAGAAAGACTAATTTGACTACTTTATTTATTTTTTAAAGTAAACTCTACACCCAAGGTGAGGCTTGAACTTAGGTCCCAGAGATCAAAAGTCACATGCTCTACTGACTGAGCCAGCCGGGGGTCCTGAATTTGACTACTTTAAAAGGAAAGCATTATTCTGTTTCTAAAAAAGCTAACTTCTCAAGACTGCTCAGGACTGAGATCAGGAAGCTTTTGTTTTTGCGGCAAAGGTATGAATGCTTAGTAATTGCACTGTGTTGTGTCACAGAAAGCAGGATAAGGGATGCTAGGGCTGTGAATGGCCACACTAATCCTTAAATCTCAGACAATACAGGACATTTCCAACAAAATTACAAAAATATTTACAGTTTGAGATGCTAAACAGACATGTTTGTATAATGATTATTTATTATATTAATCACACCAGTAGATGCCCACCTTGCTGGGTTTTTGTTGTTGTTTTTATCCCAAACTAGATCACCAAAGAGAAGAGTAGCACCTCTGTCAAAACTGGCAGTCGGATGGGTAAACTGATGTTTGAACCCCATTCTCTAAGTAGCCTCAAGAAAGGACTCGATTGCTGTGACCAGAACAGAATCCTCCGAAGAAACGTGTCACTCTTCAGAGATGGCTCCCTGCATTGCCTGGGCACTAAGGAGGAATTCGTAAAAGCCGCTCACTGTGCAGGGCCCTGGTTAGAACAGGTGGAACTTGGTGAAGGTCGCGTCCCATGGAAATTCTTACTCCCCGCCTTGCCTCCTTCACCTTGTGAGAATGAGAGCGCCTCACACCAGAAATGCTGGCGATCCCTCCGAGCAGGCTCCTGTGCTCTCTTCCCTTTAGGACAGATCTTCCTGGAGCCTTCCATTCCCTTTCCCATCTCCTACCGCCTGTGAAATTTGCTTCTAGGAGTCTGGTTTCCAGGAGCGCCTCCACCTGCCACCCCTGCACATTCCTACAGCATCTCTTAGCTTTCTGTCTAAGCCTACGAAACCCTGGCACAGCCATCCCTTTGCTGGTTGGTCCAGAGCCTCTATCAACTGGACATCCGGATATTCGAAGAGTCGGGGCCGGGGGGGGGGGGGGGGGCACGGGGGTGTGTGGCTGCAGAACAGCTCCCACTCCCAGGTCGACTCAGACCTTTTCAAGTTGCTCCGGGGCCCAGCTCACCTGCAGCGCGCAGACGGGCACCCAGTCGGCTCGGGCACCCAGAGGACCGGGACGGCCAGGCCTCTGCTCAGTCACCGACG(R_Primer_C)GGGCACGTGAGCTGCCCCTTCCCCTTTAGCGGCCGGGGCCTGCGGAGGTCTCGGTTCAGCCCCCCGCTCGCCGCAGCCGCTCCCTCCCGCAGCGACGTGATCGCCAGCGCGGCCTGGAGACCAGCGTTCTGCCTCGGGGGACACCTGAGCCCGGGGTCCCGCGTGCACACTCTCGCGCGCGGTGCGATAAACGGCAGGTGGCCCCGTGGGAGGTGCCTCTCGCGGTGCCTCTCTCTGGC

**Supplemental Methods for Genotyping Assay**

*Genotyping Assay Methods*


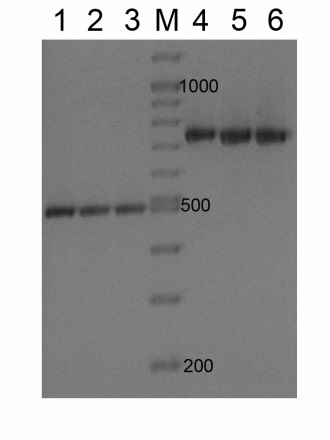
A simple PCR assay was developed for genotyping the identified deletion in WDPs using a common forward primer and two reverse primers. One reverse primer is within the deletion, and the other is downstream of the deletion. In unaffected dogs, the product is 745 bps and in affected dogs the product is 495 bps. It is possible that the unaffected dogs could produce an amplicon using both primer sets but the expected product sizes would be 745 (F_Primer_A and R_Primer_B) and ~4500 bps (F_Primer_A and R_Primer_C), but the latter band has never been observed (Figure S1).

*Genotyping Assay Results*

Once the deletion boundaries were identified a genotyping assay was developed (Figure S1). The development of a PCR based genotyping assay allowed screening of additional control Dobermans and other breeds. Dogs from eight different breeds (N=54) were tested for the mutation (Table S4). All breeds tested negative for the mutation, indicating that this deletion is not homozygous in SDPs, and not present in other breeds tested but is present in homozygous form in all WDPs, although it is possible that the mutation could exist at a frequency lower than could be detected with this sample size. The probability of this result occurring within the SDPs by chance for a non-associated variant is P <0.0001.
